# Description of a chromosomal *fosA6* tandem multicopy fragment and its *in vivo* evolution in consecutive KPC-producing *Klebsiella pneumoniae* clinical isolates

**DOI:** 10.1128/aac.01877-25

**Published:** 2026-04-20

**Authors:** María Alejandra Mateo-Vargas, Felipe Fernández-Cuenca, Lorena López-Cerero, Manuel Rodríguez-Iglesias, Fátima Galán-Sánchez

**Affiliations:** 1 EDUCA, Departamento de BiomedicinaBiotecnología y Salud Pública, Universidad de Cádiz 16727, Cádiz, Spain; 2 Instituto de Innovación e Investigación Biomédica de Cádiz (INIBICA), Hospital Universitario Puerta del Mar, Cádiz, Spain; 3 Unidad Clínica de Enfermedades Infecciosas y Microbiología, Hospital Universitario Virgen Macarena, Instituto de Biomedicina de Sevilla IBIS 273449, Seville, Spain; 4 CIBER de Enfermedades Infecciosas (CIBERINFEC), Instituto de Salud Carlos III 38176, Madrid, Spain; 5 Departamento de Microbiología, Universidad de Sevilla 16778, Seville, Spain; 6 Servicio de Microbiología, Hospital Universitario Puerta del Mar 16824, Cádiz, Spain; Universita degli Studi di Roma La Sapienza, Rome, Italy

**Keywords:** tandem multicopy, *fosA6*, fosfomycin, gene expression, *Klebsiella pneumoniae*, evolution, KPC, antimicrobial resistance

## Abstract

Chromosomal tandem duplications are scarcely reported in *Klebsiella pneumoniae*. We describe a chromosomal *fosA6* tandem multicopy fragment present at varying copy number in five consecutive *K. pneumoniae* carbapenemase-producing *K. pneumoniae* isolates recovered from a single patient over 27 months. Fosfomycin minimum inhibitory concentrations increased with copy number; however, fosA6 expression did not scale proportionally with copy number. No other fosfomycin resistance mechanism was identified. This highlights the clinical relevance of chromosomal AMR gene amplifications.

## INTRODUCTION

*Klebsiella pneumoniae* carbapenemase (KPC) is one of the most widely distributed acquired carbapenemases worldwide (1). KPC-producing *Klebsiella pneumoniae* (KPC-*Kp*) strains emerged as major hospital-associated pathogens due to their contribution to multidrug resistance. Fosfomycin, a broad-spectrum cell wall synthesis inhibitor, inactivates MurA (UDP-N-acetylglucosamine-3-enolpyruvyltransferase), disrupting the first step in peptidoglycan biosynthesis. Fosfomycin resistance may arise through defects in membrane transporters, substitutions in the MurA active site, and production of the inactivating enzyme FosA (metallo-glutathione *S*-transferase) (2, 3). Chromosomal *fosA* is present in most *K. pneumoniae* isolates (4), located downstream and overlapping the end of the putative transcriptional regulator *lysR*, whose role in the expression of *fosA* is scarcely known (5). Initially detected on *Escherichia coli* plasmids, the *fosA6* variant was associated with the *K. pneumoniae* chromosomal gene (99% similarity) (2), being later identified in KPC-*Kp* isolates (6).

To date, several cases of antibiotic-resistant gene duplications in clinical isolates have been reported (7). Although tandem multicopy of resistance genes inserted in the chromosome has been described (8), it is scarcely reported in *K. pneumoniae* (9). Moreover, there are a very small number of reports characterizing tandem duplications of *K. pneumoniae* intrinsic chromosomal genes (10). To the best of our knowledge, this is the first report describing a *fosA* tandem multicopy structure in the *K. pneumoniae* chromosome.

**Fig 1:**
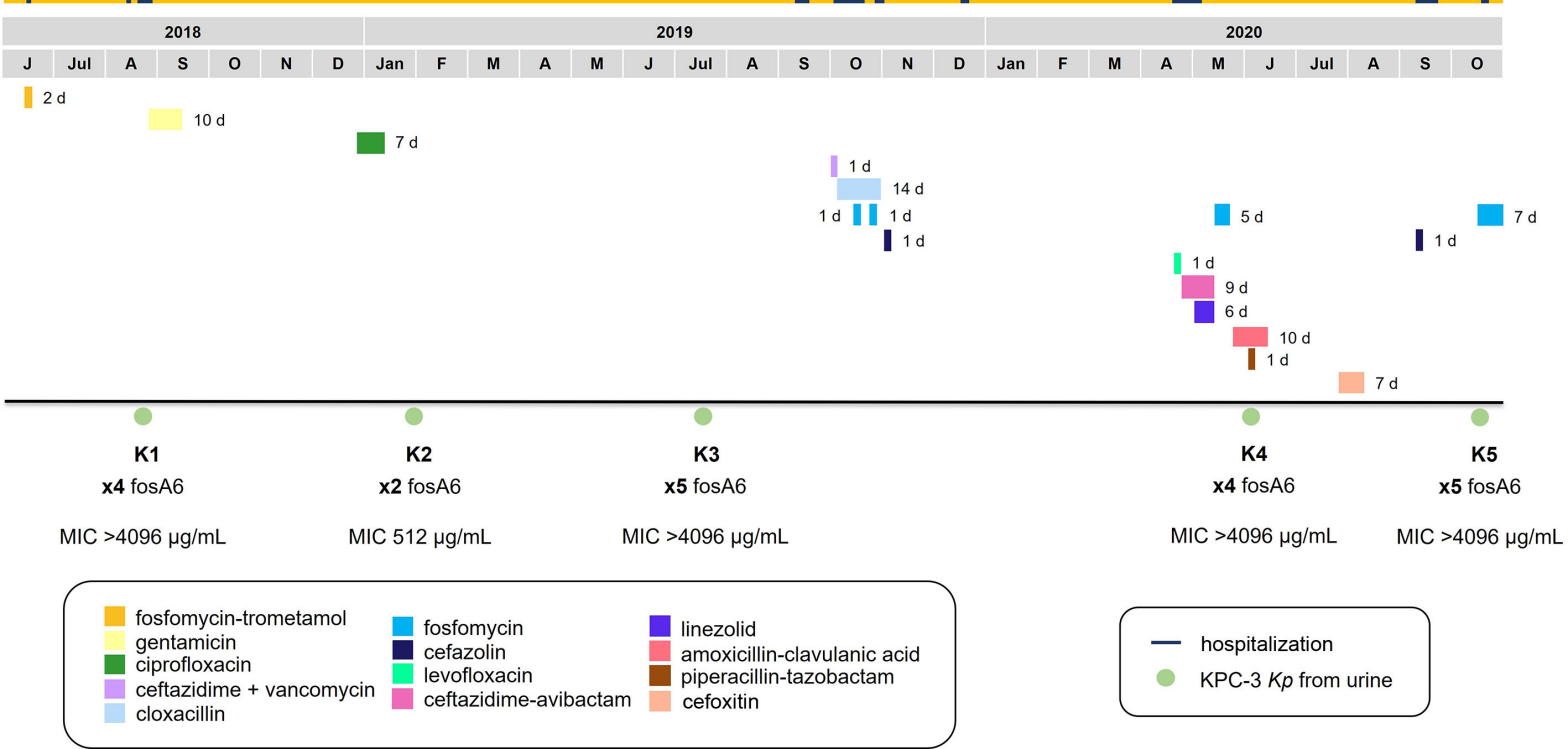
Timeline of KPC-*Kp* consecutive isolates recovered from the patient during the period 2018–2020. Antibiotic treatments are represented by color-coded bars in the same sequential order as indicated in the legend, followed by the length of the treatment (days). Blue lines on top represent the length of the hospitalizations.

Here, we report five KPC-*Kp* consecutive isolates (K1-K5) that were recovered from an 84-year-old male from August 2018 to October 2020. The patient suffered from type II diabetes mellitus and chronic end-stage renal disease. All isolates were recovered from urine samples and considered as the causative agent of urinary tract infections. They were preserved at −80°C. The patient received fosfomycin trometamol 2 months before K1 was recovered, and fosfomycin sodium 1 month before recovering K4 and 1 day before recovering K5 (Fig. 1). Minimum inhibitory concentrations (MICs) were determined for all isolates. All isolates were susceptible to meropenem-vaborbactam, imipenem-relebactam, ceftazidime-avibactam, and cefiderocol (Table 1).

**TABLE 1:** Antimicrobial susceptibility testing (MIC values and interpretation) and resistance traits of the KPC*-Kp*^*a*^

Patient	Isolate	Date of isolation	MIC (µg/mL)^*b*^											Antimicrobial resistance genes	Plasmids		
			I-R [R > 2]	MVB [R > 8]	CZA [R > 8]	FDC [R > 2]	CST [R > 2]	FOF^*c*^	AMK [R > 8]	TOB [R > 2]	FEP [R > 4]	ATM [R > 4]	TZP [R > 8]	fosA6	IncFIB(pQil)/IncFII(K)	IncFIB(K)	ColRNAI
1	K1	08/2018	2/4	0.25/8	2/4	1	16	>4,096	>32	>4	>16	>32	>32/4	(**×4**)	158,644 bp (C)	1,195 bp (L)	13,636 bp (C)
	K2	01/2019	0.5/4	0.5/8	2/4	1	8	512	32	>4	>16	>32	>32/4	(**×2**)	158,649 bp (C)	93,639 bp (C)	13,636 bp (C)
	K3	07/2019	0.5/4	0.25/8	4/4	1	8	>4,096	32	>4	>16	>32	>32/4	(**×5**)	158,645 bp (C)	129,562 bp (L)	13,636 bp (C)
	K4	06/2020	2/4	0.5/8	4/4	1	2	>4,096	32	>4	>16	>32	32/4	(**×4**)	158,647 bp (C)	93,639 bp (C)	13,637 bp (C)
	K5	10/2020	0.25/4	0.25/8	2/4	1	4	>4,096	16	>4	>16	>32	>32/4	(**×5**)	158,651 bp (C)	86,760 bp (L)	13,636 bp (C)
2	FOS-1	11/2018	0.25/4	0.25/8	2/4	1	16	32	16	>4	>16	>32	>32/4	(**×1**)	252,282 bp (C)		13,636 bp (C)

^*a*^ AMK, amikacin; ATM, aztreonam; C, circular; CST, colistin; CZA, ceftazidime–avibactam; FDC, cefiderocol; FEP, cefepime; FOF, fosfomycin; I–R, imipenem–relebactam; L, linear; MVB, meropenem–vaborbactam; TOB, tobramycin; TZP, piperacillin–tazobactam. The inhibitor was tested at a fixed concentration of 4 µg/mL for tazobactam, avibactam and relebactam, while vaborbactam was tested at 8 µg/mL. All isolates presented the antimicrobial resistance genes *bla*KPC-3, *bla*OXA-9*, *blaTEM*-1A, *blaSHV*-11, *aac(6′)-Ib, aph(3′)-Ia, aadA2, catA1*, *drfA12*, *sul1*, *qacEdelta1*, *mph(A)*, *oqxAB*, and 108_118del *mgrB*. The number of *fosA6* gene copies is shown inside brackets. *bla*OXA-9* is truncated due to a SNP at position 35A>G.

^*b*^ 2025 EUCAST Breakpoints indicated.

^*c*^ Results obtained by agar diffusion golden standard method. No breakpoint is available for non-*E. coli* Enterobacterales.

**Fig 2:**
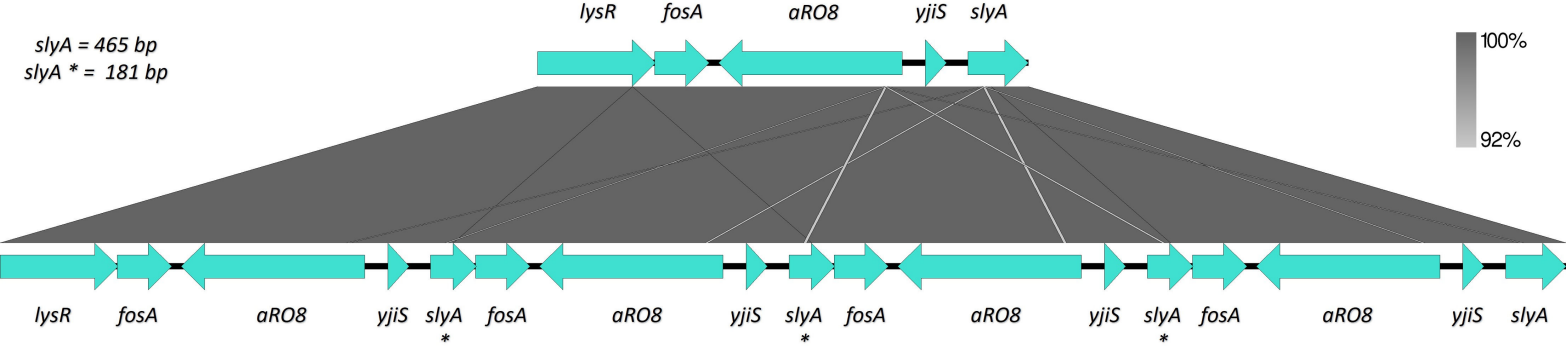
Tandem multicopy fragment genetic environment. One copy of the fragment, obtained from FOS-1 (above) is compared to the four copies fragment obtained from K1 (below). slyA* = merging of the first 181 bp of *slyA* and the last 173 bp of *lysR*.

To investigate clonal relatedness and underlying resistance mechanisms, all isolates were sequenced by MiSeq (Illumina, Inc., San Diego, USA) and Oxford Nanopore Technology (Plasmidsaurus) to obtain their hybrid assemblies. The maximum number of SNPs found among consecutive isolates was 17; therefore, they were considered clonally related (11, 12). The five isolates belonged to ST512, cgMLST3287, serotype wzi154, locus KL107, and OL2α2 (O2β). All isolates carried the same replicon sequences, distributed in three plasmids—an IncFII(K)/IncFIB(pQil) carrying the *bla*_KPC-3_ gene, an IncFIB(K), and a ColRNAI. All isolates carried the same resistance genes (Table 1), presenting more than one copy of the *fosA6* gene (Fig. 1). Genetic environment surrounding *fosA6* from all the isolates was compared through BLAST (13) and differences were visualized using Easyfig-v.2.2.5 (14). The *fosA6* gene was part of a 2,757 bp chromosomal tandem multicopy fragment that started on the last 173 bp of the *lysR* gene and included the genes *aRO8*, *yjiS*, and the first 181 bp of *slyA* (Fig. 2). All multicopy sequences were identical, including the *fosA6* gene. The copy number carried by each isolate was 4, 2, 5, 4, and 5, respectively. In light of this finding, fosfomycin susceptibility was further determined by agar dilution in Mueller-Hinton agar (OXOID) as the gold standard method, with all assays performed in technical triplicate (15). *E. coli* ATCC 25922 and *K. pneumoniae* FOS-1, a clonal related KPC-*Kp* (14 SNPs vs K1) from another patient harboring one copy of *fosA6*, were used as control strain and MIC reference, respectively. Fosfomycin MIC values of the isolates ranged from 512 to >4,096 µg/mL, with FOS-1 presenting a MIC of 32 μg/mL. The contribution of *fosA6* to these values was confirmed by the inhibition of FosA6 activity with sodium phosphonoformate using disk diffusion testing (2), since all isolates showed enlarged inhibition zones (≥5 mm) (16). Fosfomycin resistance genes *fosB*, *fosX*, *fosC*, *murA, glpT, uhpT*, *cyaA*, *crp*, *ptsI*, *uhpA*, *uhpB*, and *uhpC* (3, 17) and putative promoters P1 and P2 (5) sequences were aligned to find differences between all isolates, including FOS-1 (CLC Genomics Workbench). All genes were identical in all isolates, with P2, *fosB*, *fosX*, and *fosC* absent. All isolates carried the same SNP in P1, not affecting the −35 nor −10 boxes.

**Fig 3:**
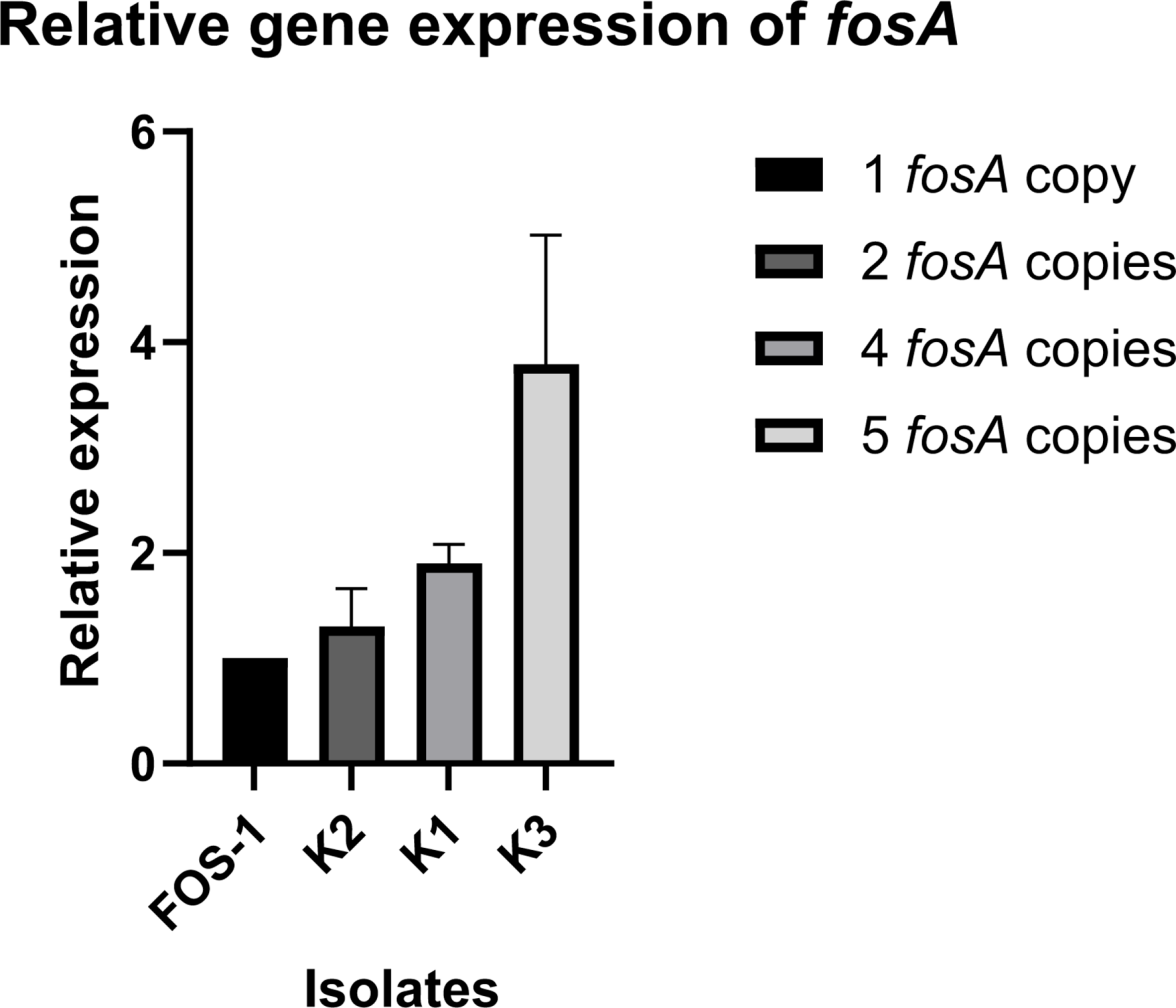
Relative gene expression of *fosA6*. Isolates are represented sequentially in ascending order of copies from left to right, setting FOS-1 expression (one copy) as 1.

To evaluate the impact of the *fosA6* copy number in gene expression, an analysis was performed for K1, K2, K3, and FOS-1 (Fig. 3). RNA was extracted using the RNeasy Mini Kit (Qiagen, Germany). After DNase treatment with Turbo DNA-free Kit (Invitrogen, Thermo Fisher Scientific), cDNA was synthesized using the Transcriptor First Strand cDNA Synthesis Kit (Roche, Switzerland). RT-qPCR was performed in a CFX96 Touch Real-Time PCR Detection System (Bio-Rad Laboratories, Inc.) with specific primers (Table S1). *rpoB* was used as a housekeeping gene to normalize gene expression. Relative expression was calculated using the 2^−△△C*t*^ method (18), setting FOS-1 expression as 1. Experiments were performed in triplicate from two independent RNA extractions. Relative gene expression of *fosA6* (presented as mean ± SD) was 1.30 ± 0.36, 1.90 ± 0.18, and 3.79 ± 1.23 times higher in K2, K1, and K3, respectively, than in FOS-1. Prior experiments showed that *fosA6* gene expression was not induced with fosfomycin exposure (data not shown).

*In vitro* experiments were performed to assess the stability of the multicopy fragments. K1, K2, and K3 were serially subcultured without antibiotic pressure, and MICs for fosfomycin were monitored (8). After 15 passages, isolates were sequenced by Nanopore technology. The multicopy fragment remained stable for every isolate.

In this study, we describe a chromosomal tandem multicopy fragment comprising the fosfomycin resistance gene *fosA6*, carried by five clonally related KPC-*Kp* isolates. To the best of our knowledge, this is the first report of a *fosA* tandem multicopy structure in any Enterobacterales.

The first isolate recovered already presented four tandem copies of this fragment, so the origin of this amplification remains unclear. Chromosomal tandem multicopy characterized *in vivo* is usually transposase-mediated (9, 19), although integrase-flanked chromosomal genes have also been found to form multimers in plasmids (8). Antibiotic selection can drive the duplication of AMR genes by intragenomic transposition (7). However, despite a prior fosfomycin exposure, no insertion sequences nor transposase-related genes in the *fosA6* genetic environment were found.

Duplicated genes usually encode functions undergoing positive selection in microbial communities, often reverting to a single-copy state in the absence of selection when recently duplicated (7). In our study, after five months without fosfomycin exposure, the copy number of this fragment was reduced from 4 (K1) to 2 (K2). However, under the same treatment-free conditions, K3 presented five copies. Tandem amplifications are intrinsically unstable and costly, leading to their loss without antibiotic pressure (20). For instance, *vanM* tandem repeats induced by vancomycin exposure in enterococci were unstable over time without antibiotic pressure (21). In other cases, such as that observed in isolates of VIM-63-producing *C. freundii*, they may remain stable after multiple subcultures without antibiotics (8). This underscores case-specific variability. In our case, the number of *fosA6* copies remained stable after 15 subcultures without antibiotic. *fosA6* seems to confer low-level fosfomycin resistance, defined as MIC < 1,024 (4) or <64 µg/mL (6), since there is no stated EUCAST MIC breakpoint for *K. pneumoniae* due to limited clinical data to support its use in monotherapy against non-*E*. *coli* Enterobacterales and the poor MIC-outcome correlation reported in some studies (22, 23). In our study, the *fosA6* copy number seems to influence fosfomycin susceptibility, as supported by the *fosA* expression analysis and FosA6 inhibition results. Although increases in gene copy number are usually proportional to their mRNA levels, gene-specific dosage compensation—where microorganisms exhibit lower mRNA expression of certain genes, presumably to reduce copy number variation gene expression costs—has been described (24), which could explain the greater fold increase observed in MICs relative to the corresponding increase in mRNA expression.

Carbapenem-resistant *K. pneumoniae* exhibiting high-level resistance to fosfomycin (MIC ≥ 1,024 µg/mL) has been described with transporter deficiencies, sometimes combined with mutations in the *fosA* gene (4). However, our isolates displayed no differences in known fosfomycin resistance genes.

Chromosomal tandem multicopy of resistance genes is scarcely reported in *K. pneumoniae*. Here, we characterize the first evidence of a *fosA* tandem multicopy fragment, whose number of copies varied among consecutive isolates. This copy variation seems to have an impact on increased MIC levels and gene expression, although it is not proportional to the copy number. Further studies are needed to evaluate the reason for this disproportionate expression.

## Data Availability

The isolates included in this study have been deposited at DDBJ/ENA/GenBank under Bioproject accession number PRJNA1096456. Hybrid assemblies of isolates K1-K5 (JBBVPW000000000, JBBVPQ000000000, JBBVPO000000000, JBBVQI000000000, and JBBVQE000000000) and FOS-1 (JBBVPR000000000).

## SUPPLEMENTAL MATERIAL

Supplemental material aac.01877-25-s0001.pdf Tables S1 and S2.

ASM does not own the copyrights to Supplemental Material that may be linked to, or accessed through, an article. The authors have granted ASM a non-exclusive, world-wide license to publish the Supplemental Material files. Please contact the corresponding author directly for reuse.
