# Supplementary material for: Description of a chromosomal fosA6 tandem multicopy fragment and its in vivo evolution in consecutive KPC-producing Klebsiella pneumoniae clinical isolates
Source: Antimicrob Agents Chemother. 2026 Apr 20;70(6):e01877-25. doi: 10.1128/aac.01877-25 (PMC13231868; doi:10.1128/aac.01877-25)
Supplement: Supplemental material — Tables S1 and S2. [file aac.01877-25-s0001.pdf]

SUPPLEMENTARY INFORMATION

Antimicrobial Susceptibility Testing

Minimum Inhibitory Concentrations (MIC) were determined for all isolates using Sensititre EUMDRXXF microtiter plates (Thermo Scientific™). MIC Test Strip (MTSTM, Liofilchem®) on Mueller-Hinton II agar plates (BD) were also used to double-check imipenem-relebactam, meropenem-vaborbactam and ceftazidime-avibactam. Cefiderocol susceptibility was assessed using 30µg disks (OXOID) and microdilution (ComASP® Cefiderocol, Liofilchem). Results were interpreted according to EUCAST v.13.1.

Genetic analysis

To investigate clonal relatedness, DNA was extracted with EZ1 Advanced XL (Qiagen). Paired-end libraries were obtained (Nextera XT DNA kit, Illumina Inc., San Diego, USA) and sequenced using 600 cycle MiSeq Reagent Kit v.3 (Illumina Inc.). Short-reads quality was evaluated (FastQC) (1) and trimming was conducted (Trimmomatic-v0.36) (2). Quality features of Illumina sequenced genomes are in **Supplementary Table 2**. All isolates were additionally sequenced using Oxford Nanopore Technology (Plasmidsaurus). Hybrid assemblies were obtained (CLC Genomic Workbench v.12.0 [QIAGEN, Les Ulis, France]) and annotated (Bakta) (3). SNPs analysis was performed (Snippy-v.4.6.0+galaxy0) with default parameters (minimum mapping quality: 60; minimum coverage: 10; minimum proportion for variant evidence: 0.9 and minimum quality in VCF column 6: 100.0), using the K1 genome as reference. Capsule types [PathogenWatch-v.12.0.4 (https://pathogen.watch/)], resistome

[ResFinder-v.4.1 (4), CARD-v.3.2.4 (5)], plasmid group assignment [PlasmidFinder-v.2.1 (6)] and IncF plasmid typing [pMLST-v.2.0 (7)] analysis were conducted. All *bla* genes, outer membrane porins genes and other antibiotic resistance genes sequences from all isolates were also aligned (CLC Genomics Workbench v.12.0).

References

1. Andrews S. 2010. FastQC: A Quality Control Tool for High Throughput Sequence Data. http://www.bioinformatics.babraham.ac.uk/projects/fastqc/.

2. Bolger AM, Lohse M, Usadel B. 2014. Trimmomatic: A flexible trimmer for Illumina sequence data. Bioinformatics 30.

3. Beyvers S, Jelonek L, Goesmann A, Schwengers O. 2025. Bakta Web – rapid and standardized genome annotation on scalable infrastructures. Nucleic Acids Res 53:W51–W56.

4. Bortolaia V, Kaas RS, Ruppe E, Roberts MC, Schwarz S, Cattoir V, Philippon A, Allesoe RL, Rebelo AR, Florensa AF, Fagelhauer L, Chakraborty T, Neumann B, Werner G, Bender JK, Stingl K, Nguyen M, Coppens J, Xavier BB, Malhotra-Kumar S, Westh H, Pinholt M, Anjum MF, Duggett NA, Kempf I, Nykäsenoja S, Olkkola S, Wieczorek K, Amaro A, Clemente L, Mossong J, Losch S, Ragimbeau C, Lund O, Aarestrup FM. 2020. ResFinder 4.0 for predictions of phenotypes from genotypes. J Antimicrob Chemother 75.

5. Alock BP, Huynh W, Chalil R, Smith KW, Raphenya AR, Wlodarski MA, Edalatmand A, Petkau A, Syed SAS, Tsang KK, Baker SJC, Dave M, McCarthy MC, Mukiri KM, Nasir JA, Golbon B, Imtiaz H, Jiang X, Kaur K, Kwong M, Liang ZCL, Niu KC, Shan P, Yang JYJ, Gray KL, Hoad GR, Jia B, Bhando T, Carfrae LA, Farha MA, French S, Gordzevich R, Rachwalski K, Tu MM, Bordeleau E, Dooley D, Griffiths E, Zubyk HL, Brown ED, Maguire F, Beiko RG, Hsiao WWL, Brinkman FSL, Domselaar G Van, McArthur AG. 2023. CARD 2023: Expanded Curation, Support for Machine Learning, and Resistome Prediction at the Comprehensive Antibiotic Resistance Database. Nucleic Acids Res.

6. Clausen PTLC, Aarestrup FM, Lund O. 2018. Rapid and precise alignment of raw reads against redundant databases with KMA. BMC Bioinformatics 19.

7. Carattoli A, Zankari E, Garciá-Fernández A, Larsen MV, Lund O, Villa L, Aarestrup FM, Hasman H. 2014. PlasmidFinder and pMLST: in silico detection and typing of plasmid. Antimicrob Agents Chemother 58.

Supplementary Table 1. Primers used in this study.

Name	*Sequence (5' - 3')*	*Tm (ºC)*	*Amplicon size (bp)*
fosA6-F	ATTTCCTCGATCCCGATGGC	60.4	96
fosA6-R	CCATCCCCTTATACGGCTGC	59.8	
rpoB-F	AAGGCGAATCCAGCTTGTTCAGC	64.3	148
rpoB-R	TGACGTTGCATGTTCGCACCCATCA	69.8	

Supplementary Table 2. Quality features of the genomes. A) Illumina sequencing. B) Nanopore sequencing

A)

Isolate	*Mean Coverage[a]*	*N50*	*Number of contigs*	*Genome size (bp)*
K1	32.6	158157	134	5559702
K2	58.3	203922	100	5574074
K3	51.4	177343	107	5572695
K4	64.9	189055	107	5565531
K5	66.5	129808	147	5554301
FOS-1	41.4	209734	101	5571037

Data obtained by Genome Assembly Report (BV-BRC: SPAdes-v3.13.0; QUAST-v5.0.2).

[a]Average short read coverage.

B)

Isolate	*Mean Coverage*	*rN50 (bp)*	*Number of contigs*	*Genome size (Mb)*
K1	45x	9149	6	5.7
K2	103x	11791	3	5.6
K3	85x	11212	4	5.7
K4	103x	11097	2	5.6
K5	100x	9726	4	5.7
FOS-1	104x	6222	4	5.6

Data obtained by Plasmidsaurus.
